# Supplementary material for: Climate-associated natural selection in the human mitochondrial genome
Source: Mol Biol Evol. 2026 Feb 17;43(5):msag044. doi: 10.1093/molbev/msag044 (PMC13134043; doi:10.1093/molbev/msag044)
Supplement: msag044_Supplementary_Data [file msag044_supplementary_data.zip › Grover-Thomas et al. - Supplementary File 2 - comparison of mean vs. centroid environmental estimates.pdf]

Supplementary File 2: Climate associated natural selection in the human mitochondrial genome.

Finley Grover-Thomas[1], Lucy van Dorp[1], Francois Balloux[1] , Aida M. Andrés[1], M. Florencia Camus[1]*

[1] Department of Genetics, Evolution and Environment, University College London, London, UK

* Corresponding author: f.camus@ucl.ac.uk; ORCID: https://orcid.org/0000-0003-0626-6865

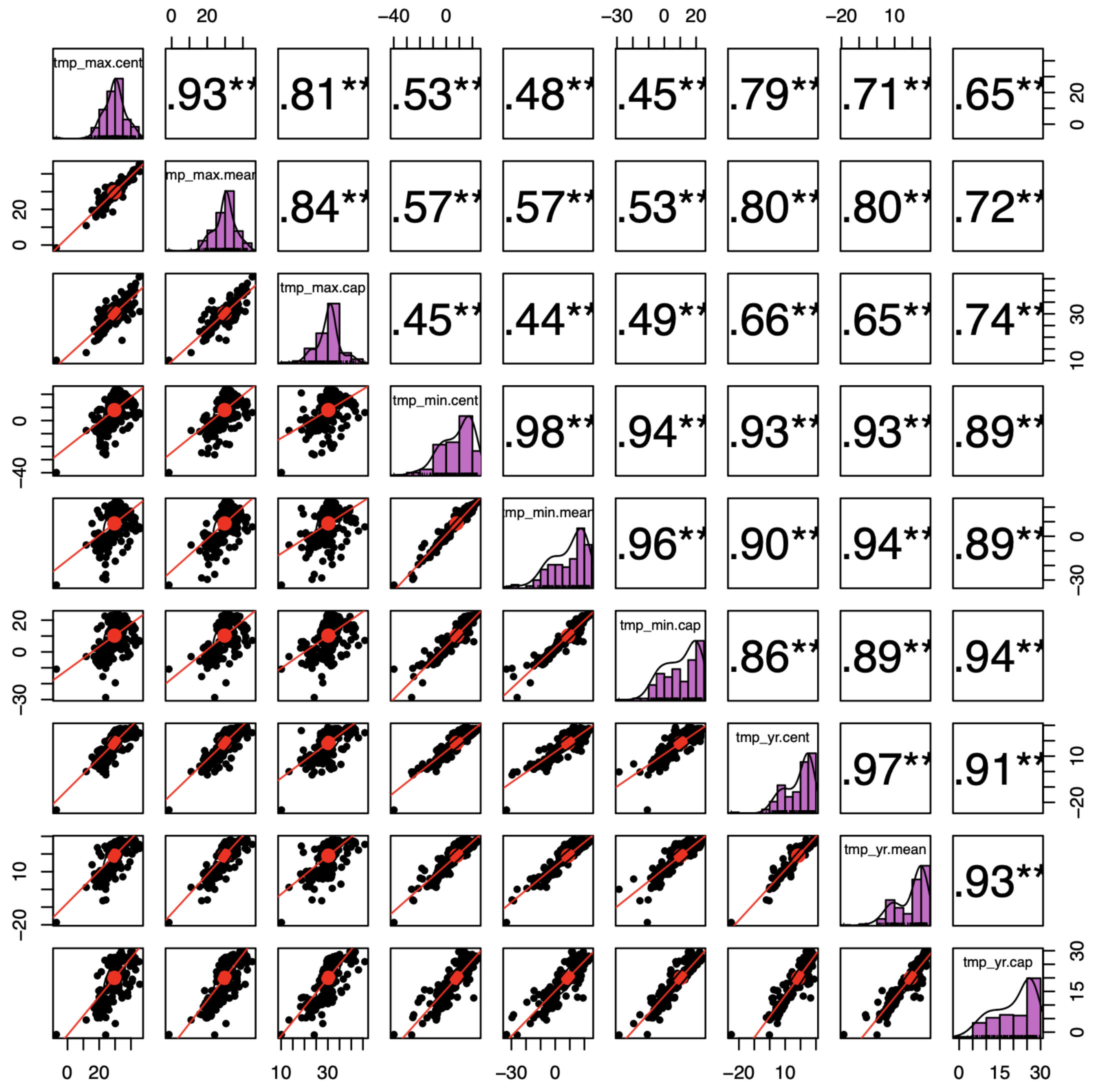

Supplementary 2 - Figure 1: **Comparison of environmental variables from mean (".mean") values vs. centroid (".cent") or capital coordinates (".cap").** Prefix denotes environmental variable. Diagonal represents histogram of a given variables' distribution. Upper triangle indicates correlation coefficients (stars denote significance), lower triangle shows scatter plots between the variables.

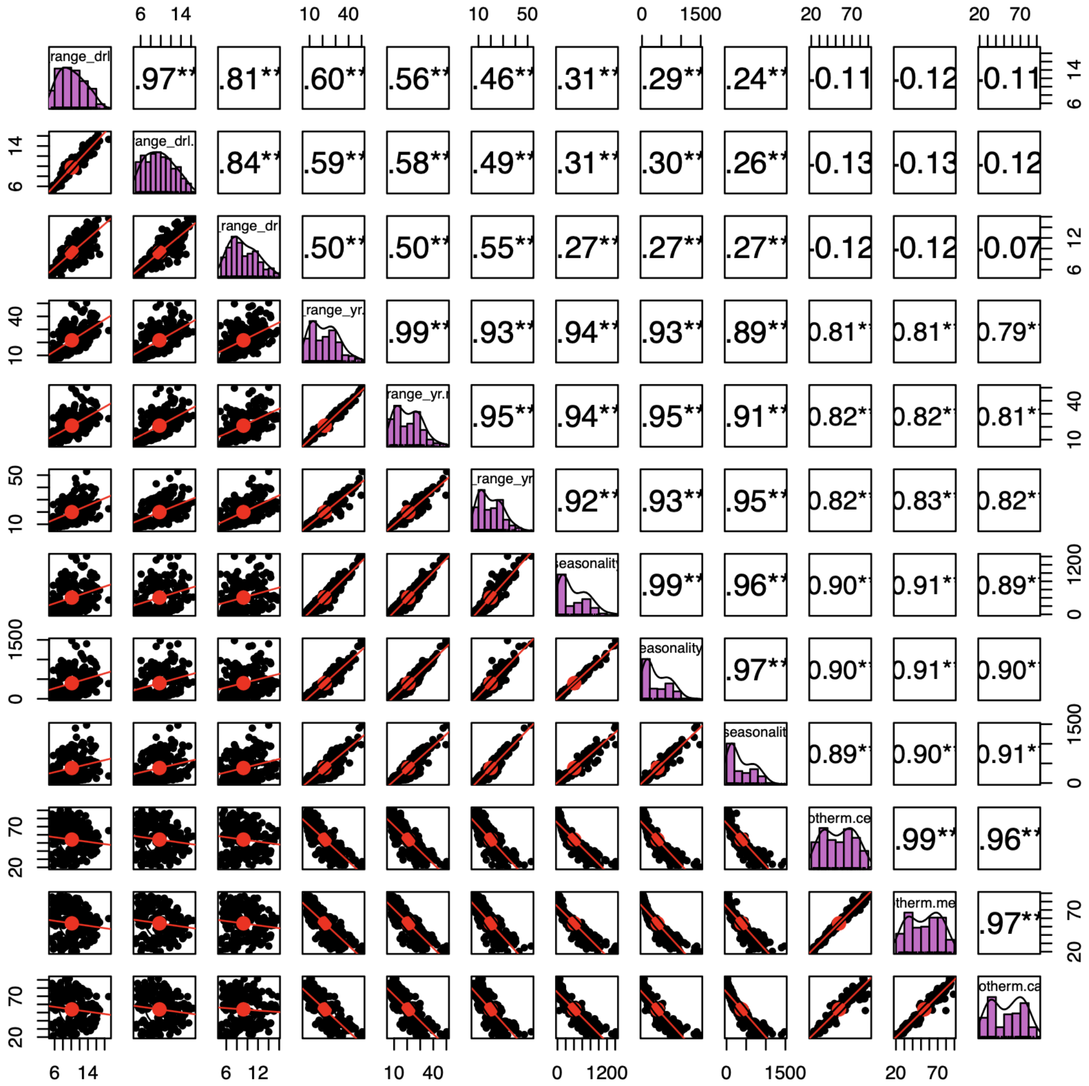

Supplementary 2 - Figure 2: **Comparison of environmental variables from mean (".mean") values vs. centroid (".cent") or capital coordinates (".cap").** Prefix denotes environmental variable. Diagonal represents histogram of a given variables' distribution. Upper triangle indicates correlation coefficients (stars denote significance), lower triangle shows scatter plots between the variables.

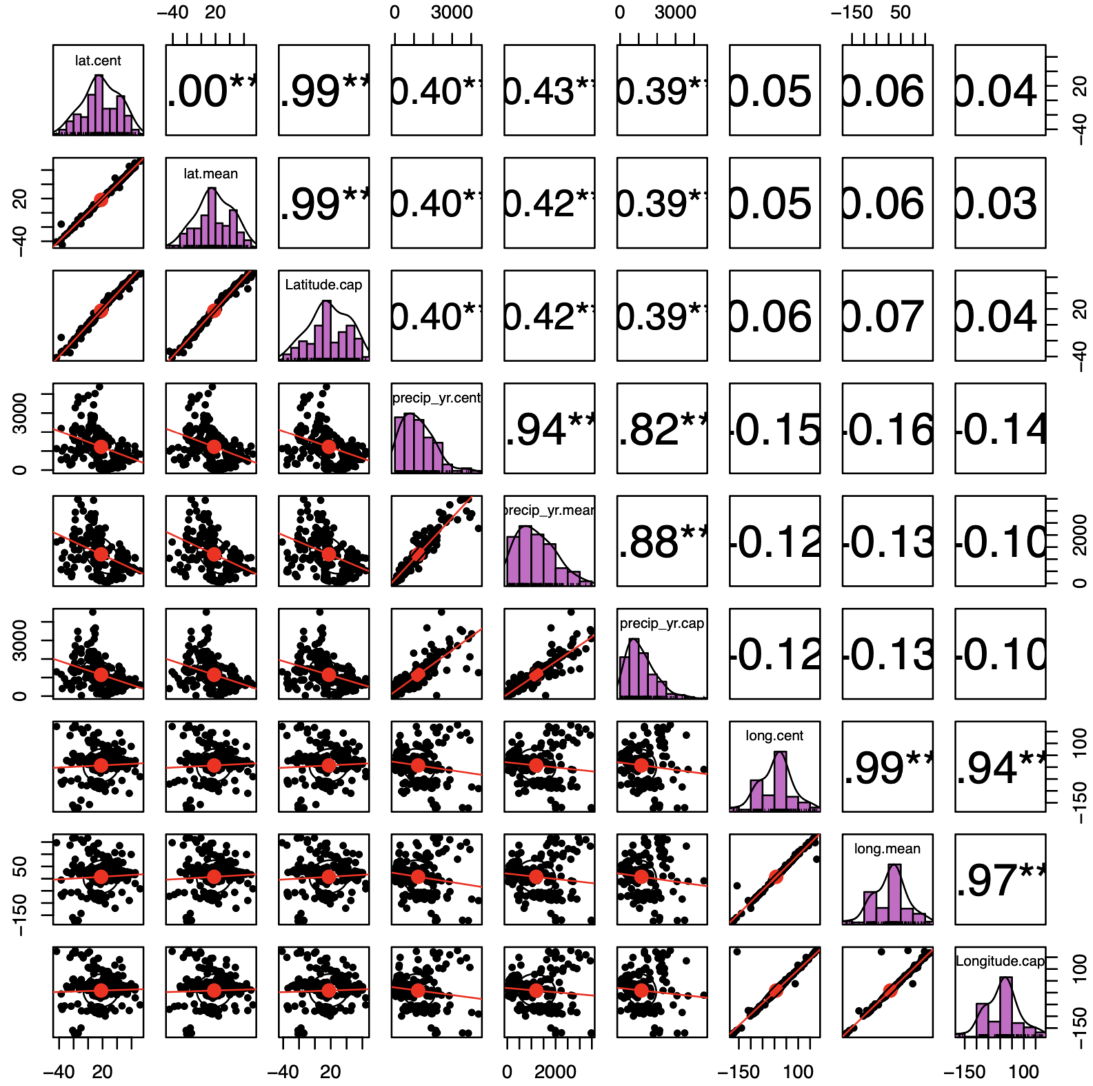

Supplementary 2 - Figure 3: **Comparison of environmental variables from mean (".mean") values vs. centroid (".cent") or capital coordinates (".cap").** Prefix denotes environmental variable. Diagonal represents histogram of a given variables' distribution. Upper triangle indicates correlation coefficients (stars denote significance), lower triangle shows scatter plots between the variables.
